# Antioxidant, Antinociceptive and CNS Activities of *Viscum orientale* and High Sensitive Quantification of Bioactive Polyphenols by UPLC

**DOI:** 10.3389/fphar.2016.00176

**Published:** 2016-06-29

**Authors:** Amina Khatun, Mahmudur Rahman, Md. Mahfizur Rahman, Hemayet Hossain, Ismet A. Jahan, Mst. Luthfun Nesa

**Affiliations:** ^1^Phytochemistry and Pharmacology Research Laboratory, Department of Pharmacy, School of Science, Engineering and Technology, Manarat International UniversityDhaka, Bangladesh; ^2^Southern Cross Plant Science, Southern Cross University, LismoreNew South Wales, Australia; ^3^Department of Pharmacy, Faculty of Health Sciences, Northern University BangladeshDhaka, Bangladesh; ^4^BCSIR Laboratories, Bangladesh Council of Scientific and Industrial ResearchDhaka, Bangladesh; ^5^Department of Pharmacy, Atish Dipankar University of Science and TechnologyDhaka, Bangladesh

**Keywords:** plant polyphenols, ultra performance liquid chromatography, antioxidant, 2, 2-diphenyl 1-picrylhydrazyl free radicals, flavonoids, total phenolic content, anti-nociceptive, neuropharmacological

## Abstract

*Viscum orientale* Willd. (Loranthaceae) has long been used in traditional medicine to treat pain, neuropharmacological disorders and various forms of tumor but not yet been reported. The aim of this study is to rationalize the traditional medicinal use of this plant by evaluating the methanol extract of *V. orientale* leaves (MEVOL) for anti-nociceptive, CNS depressant and antioxidant activities and to quantify the bioactive polyphenols present in this plant. Five polyphenolic compounds namely gallic acid, vanillic acid, caffeic acid, ellagic acid, and quercetin (17.54, 8.99, 99.61, 4523.31, and 100.15 mg/100 g of dry weight, respectively) have been identified in MEVOL using Ultra Performance Liquid Chromatography. Qualitative antioxidant activity determined by Thin Layer Chromatography indicated the presence of antioxidants. In quantitative antioxidant test using 2,2-diphenyl 1-picrylhydrazyl, MEVOL exhibited strong free antioxidant activity in a dose dependant manner (IC_50_ = 6.63 μg/ml) compared with ascorbic acid (IC_50_ = 1.91 μg/ml) and butylatedhydroxyanisole (IC_50_ = 2.27 μg/ml) controls. Total phenolic content determined using Folin Ciocaltu reagent was found to be 73.4 mg gallic acid equivalent/g of extract, while flavonoid content estimated using aluminum chloride colorimetric method was 170.7 mg quercetin equivalent/g of extract. Anti-nociceptive activity of MEVOL measured using acetic acid and formalin induced pain models in mice was significant (*p* < 0.001). MEVOL showed 65.6 and 88.8% writhing inhibition at 300 and 500 mg/kg body weight, respectively, comparing with standard diclofenac-Na (75.2% inhibition) at 25 mg/kg body weight in acetic acid induced pain model. In formalin induced pain model, paw licking was inhibited 45.93 and 56.4% in early phase and 55.66 and 72.64% in late phase at 300 and 500 mg/kg body weight, respectively, while diclofenac-Na inhibited 60.47 and 61.32% in early and late phase at 10 mg/kg body weight, respectively. In neuropharmacological activity test, overall behavioral test significantly reinforced CNS depressant activity. Spontaneous motor activities were reduced (*p* < 0.05) in both hole cross and open field tests compared with diazepam. Antioxidant activity of MEVOL is likely due to the phenolic and flavonoid compounds present within the leaf tissues. This study reveals significant *in vivo* anti-nociceptive and CNS depressant activities which justifies traditional medicinal applications of *V. orientale*.

## Introduction

Oxidative stress is caused by free radicles resulting from DNA and tissue damage, inflammation and cell death (Festa et al., 2000). OS is associated with the prevalence of cancer, cardiac diseases, neurodegenerative diseases and aging. Human physiology can neutralize OS by its own antioxidant mechanism which may fail due to an over exposure to oxidative substrate. Antioxidants neutralize free radicals and increase longevity. Vegetables and fruits are the primary dietary source of natural antioxidants which increasingly being replaced with fast food to cope up with our busy lifestyle in the recent civilizations. As a result, individuals are getting more susceptible to health problems associated with OS (Rahman et al., 2015a). Alongside, pain is a very unpleasant sensation which works as an alarm when body is recognized as an ailment state. The analgesic drug market is rapidly growing every year due to a sharp rise of pain associated patients (Rahman et al., 2016). A number of important analgesic agents have been derived from natural products including aspirin, morphine and heroin (Kumar et al., 2010). Diseases and disorders of the CNS are also some of the most common ailments distressing mankind. Approximately 32% of the population of the USA are estimated to suffer from a CNS disorder (Clement et al., 2004).

Nature is the most dependable source of potential drug substances from the beginning of history. The huge structural diversity of naturally derived molecules serves as “lead” compounds for development of new drugs to get superior pharmacological activity with lesser side effects (Muhammad et al., 2015; Rahman et al., 2015a). Therefore, there is a strong need of new antioxidants, anti-nociceptives, and CNS depressants from natural sources for the development of novel drug products.

*Viscum orientale* Willd. (Family: Loranthaceae), synonym *V. verticillatum* Roxb. locally known as Banda, is a stem-parasitic plant. Plants grow on various species of trees at low and medium altitude forests of the Chittagong hill tracts as well as the Sundarban mangrove forest in Bangladesh. It also grows in the drier regions of Sri Lanka, India and southern China and Australia (Bussing, 2000; Khare, 2008; Khatun A. et al., 2014). *V. orientale* has been described in the literature as a medicinal plant of great ethnobotanical importance (Perry and Metzger, 1980; Han et al., 1998; Bussing, 2000; Court, 2000; Sahoo, 2001; Allen and Hatfield, 2004; Trivedi, 2006; Khare, 2008). The plants parasitizing *Strychnos nux-vomica* tree are used in Indian medicine as a substitute for nux-vomica as well as for neuralgia and skin diseases (Khare, 2008). They are considered as poisonous and said to possess medicinal properties similar to those of the tree on which it grows (Kumar et al., 2013). In India, it is used for pustular itches, giddiness and stiffness (Nayak et al., 2004). In Bangladesh and the Philippines, leaf poultice is used for neuralgia (Sahoo, 2001; Medicinal Plants of Bangladesh; Philippine Medicinal Plants). Other species of *Viscum* such as *Viscum album* is reputed for its nervous system relaxing effect and believed to protect from high blood pressure and stroke. A secondary use of *V. album* has been reported for fevers, measles (to bring out the spots) and whooping cough. In Ireland and the UK, the plant has enjoyed a reputation for soothing the nerves in general and for palliating heart palpitation, epilepsy and hysteria. *V. album* combined with *Cardamine pratensis* also has a reputation for nervous aﬄictions (Allen and Hatfield, 2004).

*Viscum orientale* is reported for cytotoxic (Khatun A. et al., 2014) and antibacterial (Satish et al., 2008) activities. It is reported to contain reducing sugar, alkaloids, tannins, and flavonoids (Khatun A. et al., 2014). *V. album* has been reported for anti-tumor activity and stimulation of the immune function against several forms of cancer (Han et al., 1998; Court, 2000; Park et al., 2000; Onay-Uçar et al., 2006; Nazaruka and Orlikowskib, 2016). A wide range of biological activities have been reported including antiviral, antihypertensive, cell proliferation inhibition activities, immunomodulating, and inflammation modifying effects from other species. Medicinal preparations containing various species of *Viscum* are also listed in official pharmacopeias and used in homeopathic, phytotherapeutic, or anthroposophical remedy (Bussing, 2000; Nazaruka and Orlikowskib, 2016).

*Viscum orientale* and other species of *Viscum* are commonly used in pain, neuropharmacological disorders and various forms of tumors and cancers which are caused by free radicals where antioxidant medications are indicated. Previous studies also reported that this plant contains tannin and flavonoid type natural antioxidants. So far, this widely used plant is not scientifically evaluated for antioxidant, anti-nociceptive and CNS activities. Given *V. orientale* grows under adverse climates which may theoretically produce high levels of antioxidants and may therefore be a candidate for drug development. These backgrounds also pulled to investigate the plant for antioxidant, anti-nociceptive, and CNS potentials with advanced procedures.

## Materials and Methods

### Collection and Identification of Plant Material

*Viscum orientale* leaves were collected from the mangrove forest of Satkhira district of Bangladesh in July 2013. The parasite was collected from another mangrove tree *Exoecaria agallocha* upon which it was grown naturally. The species was taxonomically confirmed by Sarder Nasir Uddin, Principle Scientific Officer, BNH, Mirpur, Dhaka-1216, Bangladesh. The voucher specimen of the plant has been deposited and preserved in BNH library for further collection, reference and given the accession number DACB-38174.

### Chemicals

2,2-diphenyl, 1-picrylhydrazyl and BHA used in the antioxidant potential determination were obtained from Sigma Chemical Co. USA. Ascorbic acid, the standard drug in the antioxidant assay and formalin, an edematogenic agent and acetic acid, a pain inducer used in anti-nociceptive assay were collected from the Techno Drugs Limited, Bangladesh. Sodium carbonate, aluminum chloride and crystalline potassium acetate used in total phenolic and flavonoid content determination using aluminum chloride colorimetric method were purchased from Merck, Germany. GA, CH, VA, CA, EC, PCA, RH, EA, MC, KF, and QU were used as standards in UPLC analysis and purchased from Sigma–Aldrich (St. Louis, MO, USA). HPLC grade acetonitrile, methanol and acetic acid were obtained from Merck (Darmstadt, Germany). Methanol and chloroform supplied by Laboratory Patterson Scientific, UK were used as solvent.

### Instruments, Equipment, and Software

Double beam Analykjena UV visible spectrophotometer (model- Shimadzu, UV-1800, Japan), electronic balance (Sartorius balance, type BP210S; Gemini BV, Netherland), vortex mixer (VM-2000, 220 V, Digisystem laboratory instruments Inc. Taiwan) were used for this study. Chromatographic analyses using UPLC of MEVOL were carried out on a Thermo Scientific Dionex UltiMate 3000 RSLC systems (Thermo Fisher scientific inc., MA, USA). The system was coupled to a quaternary rapid separation pump (LPG-3400RS), Ultimate 3000RS auto sampler (WPS-3000) and rapid separation (DAD-3000RS). Acclaim^®^ C18 (4.6 mm × 250 mm; 5 μm) column (Dionix, USA) controlled at 30°C by a temperature controlled column compartment (TCC-3000) was used to analyze phenolic compounds. Data acquisition, peak integration and calibrations were performed with Dionix Chromeleon software (Version 6.80 RS 10).

### Test Animals

Young swiss-albino mice- *Mus musculus* of either sex of 3–4 weeks of age, weighing 20–25 g purchased from the animal research branch of the ICDDR, B were used for *in vivo* anti-nociceptive and neuropharmacological screening. The animals were housed in plastic cages (120 cm × 30 cm × 30 cm, with flake wood shavings for bedding) in standard environmental conditions at the animal house in department of Pharmacy, Manarat International University for adaptation after their purchase under standard laboratory conditions (relative humidity 55–65%, room temperature 25.0 ± 2.0°C and 12 h light–dark cycle). They were fed with ICDDR, B formulated grower pelletized standard vital feed and water was available *ad libitum* throughout the period of acclimatization.

### Ethical Statements

The research on laboratory animals in this study was carried out according to the rules governing the use of laboratory animals as acceptable internationally, comply NIH, guide for the care and use of laboratory animals and OECD guidelines and the experimental protocol was approved by the animal ethics committee, Manarat International University (Reference no: MIU/AHEC/01/2013). Adequate measures were taken for the laboratory animals to lessen suffering prior to, during and after the experiments.

### Preparation of Methanol Extract

The collected plant leaves were separated from undesirable materials, plants and plant parts and dried in open air under shade for 2 weeks. The dried leaves were ground into 40–60 mesh size, a coarse powder with the help of a suitable grinder (Capacitor start motor, Wuhu motor factory, China) wearing ear and eye protection and moving the other personnel away from the grinding premises. The leaves powder was stored in an airtight container and kept in a cool, dark, and dry place until analysis commenced. Usually the common pharmacophores present in plants are extractable with methanol, ethanol, dichloromethane, chloroform. Methanol was used as solvent in this study. 230 g of powder material of *V. orientale* was taken to a clean glass container and was soaked in 1.8 liter of methanol. The container was sealed and kept for a period 14 days accompanying occasional shaking and stirring. The whole mixture underwent a coarse filtration by a piece of clean, white, cotton material, and then was filtered through Whatman filter paper (Bibby RE200, Sterilin Ltd., UK). The obtained filtrate was concentrated at low temperature (4°C) and reduced pressure using a rotary vacuum evaporator (R-210, Buchi, Switzerland) and dried. The gummy concentrate of 10 g (4.35% yield) was obtained and designated as crude methanol extract of *V. orientale* leaves (MEVOL) (Rahman et al., 2015b). The extract was then freeze-dried with Savant Refrigerated Vapor Trap and preserved in refrigerator for further work in future.

### Experimental Procedures for Polyphenolic Compound Analysis by Using Ultra Performance Liquid Chromatography

#### Preparation of Standards and Samples

Each stock standard phenolic compound solution (100 μg/ml) was prepared in methanol by dissolving 0.005 g of the analytes into 50 ml volumetric flask. Then mixed standard solution was prepared by dilution of the stock standard solutions in methanol to give a concentration of 5 μg/ml for each polyphenols except (+) CH, CA, RH (4 μg/ml) and QU (3 μg/ml). All standard solutions to be used for at least 3 months were stored in the dark at 5°C to reduce reactions.

Methanol extract of *V. orientale* leaves solution was prepared in methanol at a concentration of 5 mg/ml by using a vortex mixer for 30 min. The sample was stored in the dark at low temperature (5°C). Spiking the sample solution with phenolic standards was done for additional identification of individual polyphenols.

All solutions (mixed standards, sample, and spiked solutions) were filtered through 0.20 μm nylon syringe filter (Sartorius, Germany) before UPLC analyses and then degassed for 15 min in an ultrasonic bath (Hwashin, Korea).

#### Conditions of Chromatogram

The phenolic composition of MEVOL was determined by a previously described UPLC protocol (Chuanphongpanich and Phanichphant, 2006) with some modification (Rahman et al., 2015b). Acetonitrile (solvent A), acetic acid solution at pH 3.0 (solvent B) and methanol (solvent C) were used as mobile phases. The system was run with the following gradient elution program: 0 min, 5%A/95%B; 10 min, 10%A/80%B/10%C; 20 min, 20%A/60%B/20%C, and 30 min, 100%A. A 5 min post run was practiced at initial conditions for equilibration of the column. 20 μl of MEVOL was injected and the flow rate was kept constant throughout the analysis at 1 ml/min. The wavelength program was optimized to monitor phenolic compounds at their respective maximum absorbance wavelengths (aaa_max_) as follows: 280 nm held for 18 min, changed to 320 nm and held for 6 min and finally changed to 380 nm for the UV detection and held for the rest of the analysis and the DAD was set at an acquisition range from 200 to 700 nm. The detection and quantification of GA, CH, VA, CA, and EC was done at 280 nm, of PCA, RH, and EA at 320 nm and of MC, QU, and KF at 380 nm.

#### Peak Analysis

Ultra performance liquid chromatography allows for highly sensitive and selective high-throughput quantification of plant antioxidant compounds (Ihara et al., 2015). The compounds were identified by comparison of the retention time, the absorbance spectrum profile of standards with each identified compound with Dionix Chromeleon software. They were also identified by running the samples after the addition of pure standards. From the calibration curves, each determined compound was quantified. The calibration curves of the standards were made by a five sets of dilution of the stock standards using methanol to yield 1.0–5.0 μg/ml for GA, VA, EC, PCA, EA, MC, and KF; 0.5–4.0 μg/ml for CH, CA, and RH, and 0.25–3.0 μg/ml for QU. The calibration curves were constructed by plotting peak area from the chromatogram versus concentration of standard with *R*^2^ more than 0.995. Data were represented as means ± SDs of triplicate independent analyses.

### Experimental Procedures for Antioxidant Potential Test

#### DPPH Radical Scavenging Activity

2,2-Diphenyl-1-picryl-hydrazyl is a relatively stable nitrogen centered free radical which produces purple color. When DPPH accepts an electron donated by an antioxidant compound, it is decolorized which can be measured by determining the absorbance (Mai and Chen, 2012).

#### Qualitative Analysis DPPH Radical Scavenging Activity

Qualitative antioxidant activity was determined by modified method of Sadhu et al. (2003) using TLC. Stock sample solutions with a concentration of 100 μg/ml were spotted on pre-coated silica gel TLC plates and the plates were developed in solvent systems of methanol:chloroform (95:5, v/v). Then they were dried at room temperature and sprayed with 0.02% DPPH in methanol. Bleaching of DPPH by the resolved bands was observed for 10 min and the color changes (yellow on purple background) were noted.

#### Quantitative Analysis for DPPH Radical Scavenging Activity

Quantitative assay was performed by the modified method of Sadhu et al. (2007). Stock solutions (10 mg/ml) of MEVOL were prepared in methanol from which serial dilutions were carried out to obtain concentrations of 5, 10, 20, 40, 60, 80, and 100 μg/ml. 2 ml of diluted solutions were added to 2 ml of a 0.004% methanol solution of DPPH, mixed and allowed to stand for 30 min for reaction. The absorbance was determined at 517 nm and from these values corresponding percentages of inhibition were calculated. Then % inhibitions were plotted against respective concentrations used and IC_50_ was calculated from the graph. The experiment was performed in triplicate and average absorbance was noted for each concentration. BHA and ascorbic acid were used as positive controls (Gupta et al., 2004). % Scavenging of the DPPH free radical was measured by using the following equation:

$$\%\;\text{Scavenging}\;\text{activity}\;\text{=}\;\frac{\text{Absorbance}\;\text{of}\;\text{the}\;\text{control-Absorbance}\;\text{of}\;\text{the}\;\text{test}\;\text{sample}}{\text{Absorbance}\;\text{of}\;\text{the}\;\text{control}}\;\times \;\text{100}.$$

#### Determination of Total Phenolic Content

The total phenolic content of MEVOL was determined on the basis of Folin–Ciocaltu method (Rahman et al., 2013). 1.0 ml of MEVOL (1 mg/ml) was mixed with 5 ml Folin–Ciocaltu reagent (1:10 v/v distilled water) and 4 ml (75 g/l) of sodium carbonate. The mixture was vortexed for 15 s and allowed to stand for 30 min at 45°C to develop the color. Then the absorbance was read at 765 nm with a spectrophotometer. The samples were prepared in triplicate for each analysis and the mean value of absorbance was obtained. The same procedure was repeated for the standard solution of GA and the calibration curve was construed. Based on the measured absorbance, the concentration of phenolics was calculated from the standard curve equation and expressed in terms of mg of gallic acid equivalent (GE)/g of dried extract).

#### Determination of Total Flavonoids

Aluminum chloride colorimetric method was used for flavonoid determination (Chang et al., 2002). MEVOL (0.5 ml of 1:10 g/ml) was mixed with 1.5 ml of methanol, 0.1 ml of 10% aluminum chloride, 0.1 ml of 1M potassium acetate and 2.8 ml of distilled water and kept at room temperature for 30 min. The absorbance of the reaction mixture was measured at 415 nm. Total flavonoid was calculated by constructing a standard curve equation and expressed as mg of QE/g of dry extract.

### Experimental Procedures for Anti-nociceptive Activity Screening

Among several methods to evaluate the anti-nociceptive activity on biological models, acetic acid induced writhing model for testing peripheral analgesic activity and formalin-induced nociception model were utilized for testing central analgesic activities. For each of two different studies, test animals were divided into control, positive control and two test groups with six mice in each group. For these analyses, MEVOL solution was prepared at the concentration of 10 mg/ml.

#### Acetic Acid Induced Writhing Method

For the anti-nociceptive activity study of MEVOL using the acetic acid induced writhing model in mice (Nesa et al., 2014). The animals of test groups received the extract at the dose of 300 and 500 mg/kg body weight. The positive control group was treated with diclofenac-Na at a dose of 25 mg/kg body weight and the control group was treated with 1% tween 80 in distilled water at a dose of 10 ml/kg body weight. Test samples, standard drug and control vehicle were administered orally 30 min before intraperitoneal administration of 0.7% (v/v) acetic acid solution (0.1 ml/10 g body weight) to induce abdominal contractions or writhing. After 5 min of acetic acid administration, the number of writhing (constriction of abdomen, turning of trunk and extension of hind legs) for each animal was counted for 15 min. The number of writhing in the control was considered as 100% and percent inhibition was calculated as follows:

$$\%\;\text{Inhibition}\;\text{of}\;\text{writhing}\;\text{=}\;\text{1-}\frac{\text{Treated}\;\text{mean}}{\text{Control}\;\text{mean}}\;\times \;\text{100}$$

#### Formalin Induced Paw Licking in Mice

In the anti-nociceptive activity test using the formalin induced paw licking, the edematogenic agent, formalin solution (0.2 ml of 5% v/v) was freshly prepared in distilled water (Nesa et al., 2014). 20 μl of 5% formalin was injected into the dorsal surface of the right hind paw 60 min after administration of MEVOL solution (300 and 500 mg/ kg body weight) to the test groups’ mice and 30 min after administration of diclofenac-Na (10 mg/kg body weight) to the positive control mice. Control group received 5% formalin. The mice were observed for 30 min after the injection of formalin. The first 5 min post formalin injection was referred to as the early phase and the period between 15 and 30 min as the late phase. The total time spent licking or biting the injured paw (pain behavior) was measured with a stop watch.

### Experimental Procedures for CNS Activity Test

#### Behavioral Profile in Mice

To evaluate the general behavioral profile, the effects MEVOL at the dose of 0.01, 0.05, and 0.10 g/kg body weight on mice were compared with pentonarbitone at the dose of 0.03 g/kg body weight according to the conventional methods with some modification (Turner, 1965).

#### Open Field Test and Hole Cross Test

Open field and hole cross tests were carried out with some modification of the method of Ahmed et al. (Ahmed et al., 2008; Nesa et al., 2014). In both tests, animals were divided into control, standard and two test groups (*n* = 6 per group). The control group received vehicle (1% tween 80 in water at the dose of 10 ml/kg). The test groups received the MEVOL at the doses of 300 and 500 mg/kg body weight and standard group received diazepam at the dose of 1 mg/kg body weight orally. In the open field test, the animals were placed on the floor of an open field (100 × 100 × 40 cm) divided into a series of squares. The number of squares visited by each animal was counted for 3 min on 0, 30, 60, 90, and 120 min during the study period. In hole cross test, each animal was then placed in one side of the chamber and the number of passages of each animal through the hole from one chamber to another was recorded for 3 min on 0, 30, 60, 90, and 120 min during the study period.

### Statistical Analysis

All values obtained from animal model studies were reported as mean ± SD (Lopes et al., 2012) of six replicates. Data analysis was conceded using Microsoft Excel, Microsoft Corporation, USA, to attain the descriptive statistics. The different levels of significances within the separate treated groups were analyzed using one-way analysis of variance (Denev et al., 2009) followed by Dunnett’s test to determine statistical significance. Statistical differences between control and treated groups were tested by Student’s *t*-test. Variances with *p* < 0.05 were considered statistically significant.

## Results And Discussion

**FIGURE 1:**
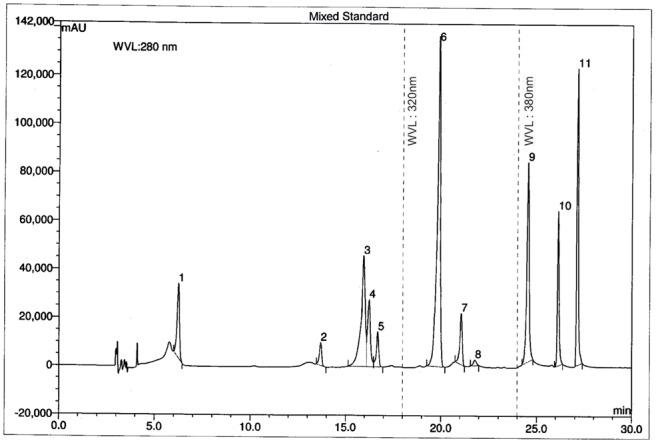
**Ultra performance liquid chromatography chromatogram of a standard mixture of polyphenolic compounds.** Peaks: 1, gallic acid; 2, (+)-catechin; 3, vanillic acid; 4, caffeic acid; 5, (–)-epicatechin; 6, p-coumaric acid; 7, rutin hydrate; 8, ellagic acid; 9, myricetin; 10, quercetin; 11, kaempferol.

**FIGURE 2:**
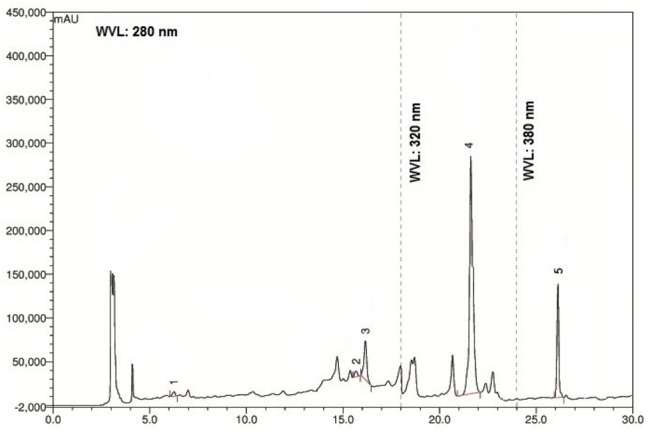
**Ultra performance liquid chromatography chromatogram of methanol extract of *V. orientale* Willd.** Peaks: 1, gallic acid; 2, vanillic acid; 3, caffeic acid; 4, ellagic acid; 5, quercetin.

The chromatographic separations of the polyphenols of the standard mixture and MEVOL were shown in **Figures 1** and **2**, respectively, and the content of each phenolic compound was presented as the mean of three determinations (**Table 1**). The experimental results indicate the presence of an especially high concentration of CA, EA, and QU (QA) of 99.61, 4523.31, and 100.15 mg per 100 g of dry MEVOL weight, respectively. GA and VA are detected in the extract but in lower amount (17.54 and 8.99 mg per 100 g of dry MEVOL weight, respectively).

**Table 1:** Polyphenolic compounds in the MEVOL.

Polyphenolic compounds	Mixed standards		MEVOL		Content (mg/100 g of dry extract)	% RSD
	Conc^n^ (μg/ml)	Avg. area	Conc^n^ (μg/ml)	Avg. area		
GA	5.0	4538.20	5000	804.09	17.54	0.34
CH	4.0	1390.19		–		
VA	5.0	11479.90		1043.28	8.99	0.18
CA	4.0	4910.55		6175.85	99.61	1.92
EC	5.0	2152.05		–		
PCA	5.0	26059.00		–		
RH	4.0	3033.85		–		
EA	5.0	424.20		19781.07	4523.31	5.73
MC	5.0	11393.0		–		
QU	3.0	6330.56		10673.07	100.15	2.58
KF	5.0	11342.70		–		

*Conc^*n*^, Concentration; Avg., Average; *n* = 3; MEVOL, Methanol extract of *Viscum orientale* leaves; RSD, Relative standard deviation; GA, gallic acid; CH, (+)-catechin hydrate; VA, vanillic acid; CA, caffeic acid; EC, (-)-epicatechin; PCA, *p*-coumaric acid; RH, rutin hydrate; EA, ellagic acid; MC, myricetin; QU, quercetin; KF, kaempferol.*

MEVOL was evaluated for *in vitro* antioxidant activity using DPPH. MEVOL showed very significant antioxidant activity (IC_50_ = 6.63 μg/ml) comparing with standard ascorbic acid (IC_50_ = 1.91 μg/ml) and BHA (2.27 μg/ml). This activity was increased with the concentration of MEVOL.

The total phenolic content in MEVOL using the Folin-Ciocalteu’s reagent was expressed in terms of GE/g of dried extract. The equation obtained from standard curve was: *y* = 6.9103x – 0.0936, *R*^2^ = 0.9972. The total phenolic content was 73.4 mg GE/g of dried MEVOL.

Flavonoid content in MEVOL determined spectro-photometrically was expressed in terms of mg QE/g of dried extract (the standard curve equation: *y* = 4.738x + 0.0355, *R*^2^ = 0.999). Flavonoid content was found 170.6 mg QE/g of dried MEVOL.

Overproduction of ROS causes diseases like type II diabetes, cardiovascular diseases, vascular, and neural diseases and reproductive dysfunctions (Kajiya et al., 2004) and decreases efficiency of antioxidant defenses (Rahimi et al., 2005; Amaral et al., 2008). In this respect, polyphenolic compounds like flavonoids and phenolic acids are of interest because of their potent antioxidant activity in biological system (Blokhina et al., 2003; Pandey and Rizvi, 2009). Recently, it has been reported that VA and QU inhibit OS (Zhang et al., 2011; Calixto-Campos et al., 2015a). GA down regulates the reactive species generation and enhances the ratio of reduced glutathione/oxidized glutathione (Kim, 2007). CA has DPPH scavenging activity (Gülçin, 2006). EA is reputed to reduce H_2_O_2_ (Festa et al., 2000). In most cases, antiradical plant phenolic constituents may synergistically enhance the antioxidant activity to each other (Iacopini et al., 2008). Many studies support the use of these active phytochemicals as anti-inflammatory, anti-oxidants, anti-cancer agent, and against liver injury, which may act by preventing the ROS, lipid peroxidation and augmenting the antioxidant defense system (Lotfi, 2011). The experimental plant extract was found enriched with GA, CA, VA, EA, and QU (**Table 1**). This may significant increase anti-oxidant activity. Another explanation for this high antioxidant property may be due to the antioxidant activity of the host plant *Excoecaria agallocha* (Masuda et al., 1999; Konishi et al., 2003).

**FIGURE 3:**
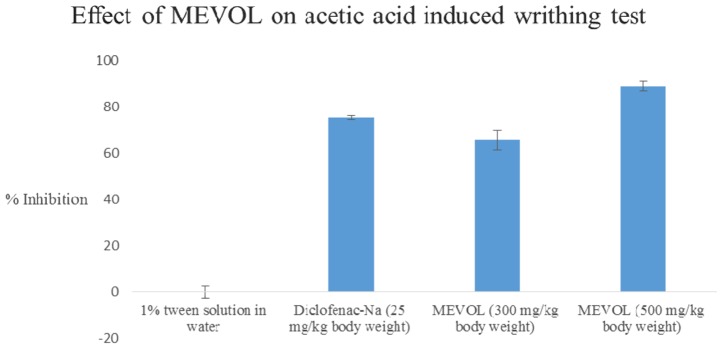
**Effect of the MEVOL on acetic acid induced writhing of mice.** *n* = 6; here, *p* < 0.001; Data are represented as mean ± SD; MEVOL, Methanol extract of *Viscum orientale* leaves.

**FIGURE 4:**
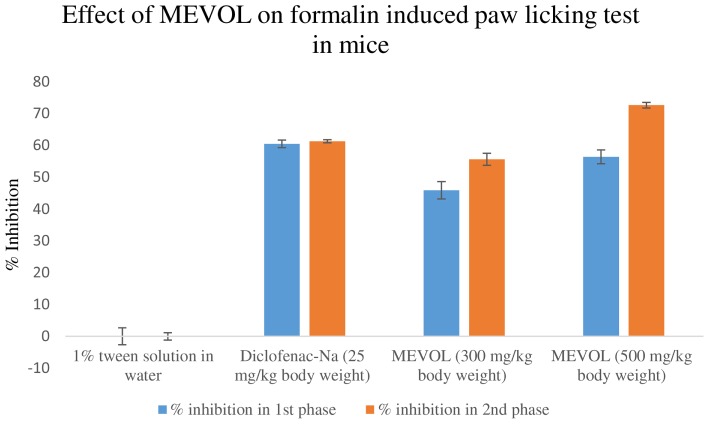
**Effect of the methanol extract of *Viscum orientale* leaves on formalin induced paw licking test in mice.** *n* = 6; here, *p* < 0.001. Data are represented as mean ± SD; MEVOL, Methanol extract of *Viscum orientale* leaves.

In the acetic acid induced writhing test, MEVOL produced 65.6 and 88.8% of writhing inhibition at 300 and 500 mg/kg of body weight, respectively, whereas the counterpart standard diclofenac-Na produced 75.2% of writhing inhibition at 25 mg/kg body weight in test animals. Both occurred in a dose dependant and time dependant manner (**Figure 3**). In the formalin-induced pain model in mice, MEVOL significantly (*p* < 0.001) suppressed the licking activity in either phase, especially at the late phase in both a dose dependant and time dependant manner. Inhibitions were found 45.93, 56.40, and 60.47% in early phase and 55.66, 72.64, and 61.32% in late phase at 300 and 500 mg/kg of body weight MEVOL and standard diclofenac-Na at the dose of 10 mg/kg body weight, respectively, (**Figure 4**).

The acetic acid induced writhing method is an analgesic-mediated behavioral observation method where intraperitoneal administration of acetic acid increases prostaglandin E2 and F2α levels in the peritoneal fluid (Ahmed et al., 2008). Any agent that lowers the number of writhings demonstrates analgesia, by inhibiting prostaglandin synthesis, a peripheral mechanism of pain inhibition. On the other hand, a formalin model normally postulates the site and the mechanism of action of the anti-nociception. This biphasic model is represented by neurogenic (0–5 min) and inflammatory pain (15–30 min), respectively. Narcotics like steroids inhibit both while NSAIDs suppress mainly the late phase (Khatun H. et al., 2014).

Bioflavonoids and polyphenolics were found to exhibit anti-nociceptive activity in chemical induced nociception, including formalin and acetic acid (Leal et al., 2011; Lopes et al., 2012; Morucci et al., 2012). The polyphenolics found in this study like GA, CA, VA, and QU directly inhibit neuro-inflammatory pain (Jager and Saaby, 2011; Farbood et al., 2013; Trevisan et al., 2014; Calixto-Campos et al., 2015a). EA was found to agonize the anti-nociceptive action of the serotonin-norepinephrine reuptake inhibitors in mouse and synergize the anti-nociceptive and anti-inflammatory effect through the central and peripheral sites of action (Mansouri et al., 2015). VA were reported as the active constituent in chemical nociceptive models in acetic acid induced writhing model and in a formalin induced paw licking model, producing the inhibition of the writhing and licking responses (Morucci et al., 2012). Antinociceptive effect of VA occurs by a mechanism partly dependent upon the opioid system, while the anti-inflammatory action was manifested in inflammatory processes dependent on polymorphonuclear cells and are probably related to the VA inhibition of cytokines (Leal et al., 2011). QU decreases the pain threshold level (Rylski et al., 1979). MEVOL was found to contain VA and QU which may be responsible for its anti-nociceptive activity.

Gallic acid was found to reduce pain and edema in inflammatory and neurogenic pain model. It was revealed as potential ankyrin 1 receptor antagonist with anti-nociceptive activity in related models of clinical pain without noticeable side effects (Trevisan et al., 2014). It was also reported to exhibit cerebro-protective properties by enhancing the antioxidant defense against ischemia/reperfusion induced brain injuries in rats (Farbood et al., 2013). VA exerts anti-inflammatory action by inhibiting oxidative stress, pro-inflammatory cytokine production, and transcriptional factor-NFκB activation. Its mechanisms of action involves antioxidant effects and NFκB-related inhibition of pro-inflammatory cytokine production (Calixto-Campos et al., 2015a). CA esterifies into CA phenethyl ester and produce AMP kinase (AMPKα), erythropoietin (EPO), and heme-oxygenase-1 (HO-1) which mediate anti-neuro-inflammatory response in microglial cells (Tsai et al., 2015). QU was proven to reduce the Ehrlich tumor-induced cancer pain by decreasing the formation of hyper-algesic cytokines, neutrophil recruitment, and oxidative stress as well as by activating an opioid-dependent analgesic pathway and potentiation of morphine analgesia (Calixto-Campos et al., 2015b).

In this study, MEVOL caused both dose and time-dependent antinociception against chemical induced nociception (pain) in mice. The extract showed significant effect in these pain inductions and suggests that the analgesic effect of MEVOL may in part be related to its anti-inflammatory and neurogenic pain (Nwafor and Okwuasaba, 2003).

The CNS activity was evaluated by observing the reduction of locomotor and exploratory activities in the open field and hole cross tests. The extract exhibited CNS depressant activity by decreasing exploratory behavior in mice. Moreover, the study of locomotor activity showed that the MEVOL decreased the frequency and the amplitude of movements. Since, locomotor activity is a measure of the level of excitability of the CNS, this decrease in spontaneous motor activity could be attributed to CNS depressing effect of the plant extracts (Nesa et al., 2014).

**FIGURE 5:**
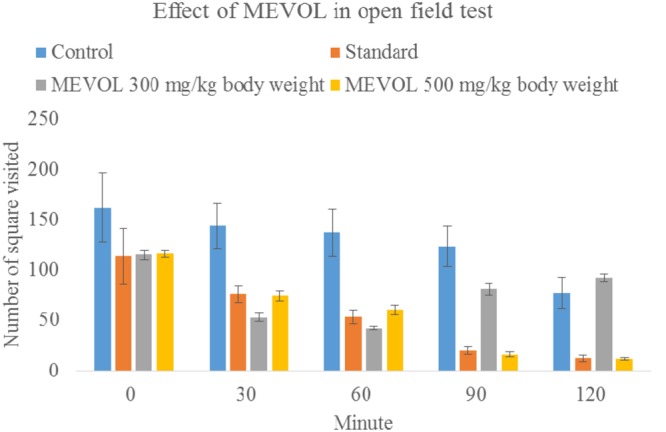
**Effect of MEVOL on mice in open field test.** *n* = 6; Data are represented as mean ± SD; Standard, Diazepam 1 mg/kg body weight; MEVOL, Methanol extract of *Viscum orientale* leaves.

**FIGURE 6:**
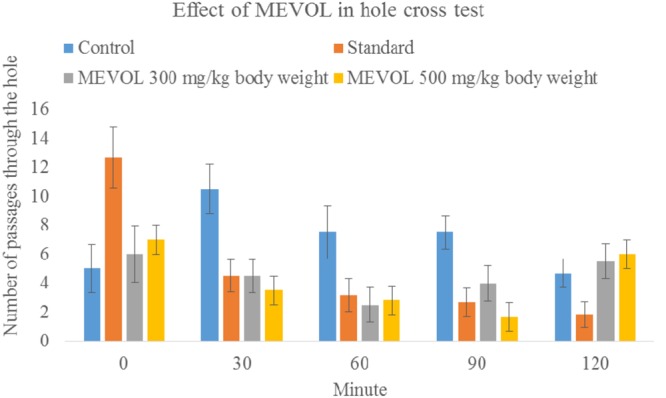
**Effect of the MEVOL on mice in hole cross test.** *n* = 6; Data are represented as meanś standard deviation; Standard = Diazepam 1 mg/kg body 2 weight; MEVOL = Methanol extract of *Viscum orientale leaves*.

As *V. orientale* has long been used traditionally to treat neuropharmacological disorders, behavioral profile was examined along with open-field test and hole cross test in mice to evaluate the neuropharmacological activity. The MEVOL induced CNS depressing activities are comparable with standard pentonarbitone. The extract at 0.10 g/kg body weight showed the highest depression (**Table 2**). In the open-field test and hole cross test, MEVOL exhibited a decrease in the movements of the test animals at all dose levels. The results were statistically significant for all doses with a non dose-dependent response (**Figures 5** and **6**).

**Table 2:** Effect of the MEVOL on behavioral profile in mice.

Treatment	Dose (g/kg)	Awareness	Touch response	Pain response	Righting reflex	Pinna strength	Mortality
Control (Tween-80)	5	0	0	0	0	0	0
Pentobarbitone	0.03	4+	4+	4+	4+	4+	0
	0.01	2+	3+	3+	3+	2+	0
MEVOL	0.05	3+	3+	3+	3+	3+	0
	0.10	4+	3+	4+	4+	3+	0

*MEVOL, Methanol extract of *Viscum orientale* leaves; Key for scoring of depression: 0, no effect (normal); +, slight; 2+, moderate; 3+, strong; 4+, very strong.*

Research has shown that plants containing flavonoids, saponins, and tannins are useful in many CNS disorders (Nwafor and Okwuasaba, 2003; Chirumbolo, 2011; Rahman et al., 2015b). Gamma-aminobutyric acid (GABA) is the major inhibitory neurotransmitter in the CNS. Earlier investigations suggest that many flavonoids and neuroactive steroids were found to be a ligand for the GABA-A receptors in the CNS; which led to the assumption that they can act as benzodiazepine (GABA agonist) like molecules (Nesa et al., 2014). GA possesses anxiolytic activity and prevents the apoptotic death of cortical neurons *in vitro* by inhibiting amyloid beta induced glutamate release and the generation of ROS. GA protects and improves neuron function in critical brain regions involved with cognition after cerebral damage by ischemia (Farbood et al., 2013). Activities of EA are linked with protection of neuronal abnormalities like anti-depressant, anti-anxiety, anti-nociception. EA inhibits secretase enzymatic activities in brain, thus prevents the main pathologic hallmark of Alzheimer’s disease (Mehan et al., 2015). EA has a major neuroprotective role in dementia (Kaur et al., 2015). QU has been traditionally used as a nerve calming medicine and was found to possess selective MAO-A and MAO-B inhibitory activity. QU also showed ameliorating effects both on the central and peripheral nervous system by promoting the functional recovery of spinal cord injury (Rahman et al., 2014). Tannins and flavonoids found in MEVOL may be responsible for CNS depressant activity. The extract possibly reduce anxiety in animal models and more selective compounds can be identified further from the plant (Chirumbolo, 2011).

## Conclusion

This study revealed the potential antioxidant, anti-nociceptive and CNS depressant activities of MEVOL. The enriched polyphenols and flavonoid composition of MEVOL is associated with the world wide ethnobotanical use of this plant in the treatment of pain and neurogenic disorders. *V. orientale* contains a wide varieties of antioxidants which may have different mechnisms of action. MEVOL also contains high amount of polyphenolic compounds which may be effective against various tumors, cancers and immunodisorders. MEVOL was found to have both peripheral and neurogenic anti-nociceptive and CNS depressant activities in different experiment models. Further analysis is warranted to investigate more precise modes of action of MEVOL and to study it at lower concentrations, which would be therapeutically more appropriate. The characterisation of secondary metabolites from *V. orientale* may be utilized in future for the development of novel drug products to treat non-communicable diseases.

## Author Contributions

AK and MR equally contributed in perceiving and designing the study. MMR extracted and prepared the plant for the study. AK, MR, HH, IAJ, and MLN performed the experiments and collected data. Data analysis and draft of the manuscript were completed by AK and MR. All the authors revised and approved the content of manuscript.

## Conflict of Interest Statement

The authors declare that the research was conducted in the absence of any commercial or financial relationships that could be construed as a potential conflict of interest.

## Abbreviations

BHA: butylatedhydroxyanisole
BNH: bangladesh national herbarium
CA: caffeic acid
CH: (+)-catechin hydrate
CNS: central nervous system
DAD: diode array detector
DPPH: 2,2-Diphenyl-1-picryl-hydrazyl
EA: ellagic acid
EC: (-)-epicatechin
GA: gallic acid
ICDDR: B, international center for diarrhoeal disease and research, Bangladesh
KF: kaempferol
MC: myricetin
MEVOL: methanol extract of *Viscum orientale* leaves
NIH: national institutes of health
OECD: organization for economic co-operation and development
OS: oxidative stress
PCA: *p*-Coumaric acid
QE: quercetin equivalent
QU: quercetin
RH: rutin hydrate
ROS: reactive oxygen species
RSLC: rapid separation liquid chromatography
TLC: thin layer chromatography
UPLC: ultra performance liquid chromatography
UV: ultra violet
*V. orientale*: *Viscum orientale*
VA: vanillic acid
